# Tasks for artificial intelligence in prostate MRI

**DOI:** 10.1186/s41747-022-00287-9

**Published:** 2022-07-31

**Authors:** Mason J. Belue, Baris Turkbey

**Affiliations:** Molecular Imaging Branch, National Cancer Institute, National Institutes of Health Bethesda, 10 Center Dr., MSC 1182, Building 10, Room B3B85, Bethesda, MD 20892-1088 USA

**Keywords:** Artificial intelligence, Deep learning, Machine learning, Magnetic resonance imaging, Prostatic neoplasms

## Abstract

The advent of precision medicine, increasing clinical needs, and imaging availability among many other factors in the prostate cancer diagnostic pathway has engendered the utilization of artificial intelligence (AI). AI carries a vast number of potential applications in every step of the prostate cancer diagnostic pathway from classifying/improving prostate multiparametric magnetic resonance image quality, prostate segmentation, anatomically segmenting cancer suspicious foci, detecting and differentiating clinically insignificant cancers from clinically significant cancers on a voxel-level, and classifying entire lesions into Prostate Imaging Reporting and Data System categories/Gleason scores. Multiple studies in all these areas have shown many promising results approximating accuracies of radiologists. Despite this flourishing research, more prospective multicenter studies are needed to uncover the full impact and utility of AI on improving radiologist performance and clinical management of prostate cancer. In this narrative review, we aim to introduce emerging medical imaging AI paper quality metrics such as the Checklist for Artificial Intelligence in Medical Imaging (CLAIM) and Field-Weighted Citation Impact (FWCI), dive into some of the top AI models for segmentation, detection, and classification.

## Key points

- Artificial intelligence (AI) offers potential applications for various steps of prostate magnetic resonance imaging workflow.
- Prostate segmentation, intraprostatic lesion detection, and classification AI tools are commonly reported in the literature with promising results.
- Prospective multicenter studies are needed to determine impact of AI on improving radiologist performance.

## Background

Artificial Intelligence (AI) is an umbrella term that encompasses both machine learning (ML) and deep learning (DL). Traditional ML methods usually require several preprocessing steps which include anatomical segmentation and feature extraction whereas DL is a subfield of ML that does not necessarily depend on hand-crafted features and independently identifies features to generate desired output predictions [1, 2]. DL, more commonly than ML, makes use of artificial neural networks that use statistical models inspired and partially modeled on biological neural networks. The use of artificial neural networks allows for the approximation of nonlinear relationships between the inputs and outputs [3]. A major challenge for prostate cancer (PCa) management is the lack of non-invasive tools that can differentiate clinically significant PCa (csPCa) and clinically insignificant PCa (cisPCa), resulting in overdiagnosis and overtreatment. There are many different definitions of csPCa ranging from Gleason score ≥ 6 or ≥ 7 permutated with various clinical factors including prostate-specific antigen (PSA) cutoffs, presence of extra-prostatic extension, and biopsy-core cancer percentage [4]. The most common definition of csPCa across most studies is Gleason score ≥ 7.

Many challenges and potential improvements remain in the prostate cancer diagnostic pathway that may be addressed by AI with the common goal of potentially reducing cisPCa overdiagnosis and csPCa underdiagnosis. AI may help accomplish improved cancer detection and/or classification across benign and malignant entities and it may aid in segmentation of suspicious foci and normal anatomy on magnetic resonance imaging (MRI) scans for tasks such as volume estimation or treatment planning utilizing transrectal ultrasound-guided biopsy [5]. AI can also help with the initial evaluation or triaging of prostate multiparametric MRI (mpMRI) cases (*i.e*., picking/identifying prostate MRI examinations with more atypical image characteristics) and of image quality (*i.e*., classifying mpMRI scans as diagnostic *versus* nondiagnostic) [6–8]. All these steps in the PCa diagnostic pathway may suffer from low inter-reader agreement of various sources which AI may also be able to improve upon. Once clinical efficacy of AI systems is demonstrated, clinical deployment can be envisioned as a companion system that creates attention boxes/maps for the radiologist during their clinical read, serves as a second reader providing independent diagnoses, or can be utilized as patient triage systems [9].

**Fig. 1:**
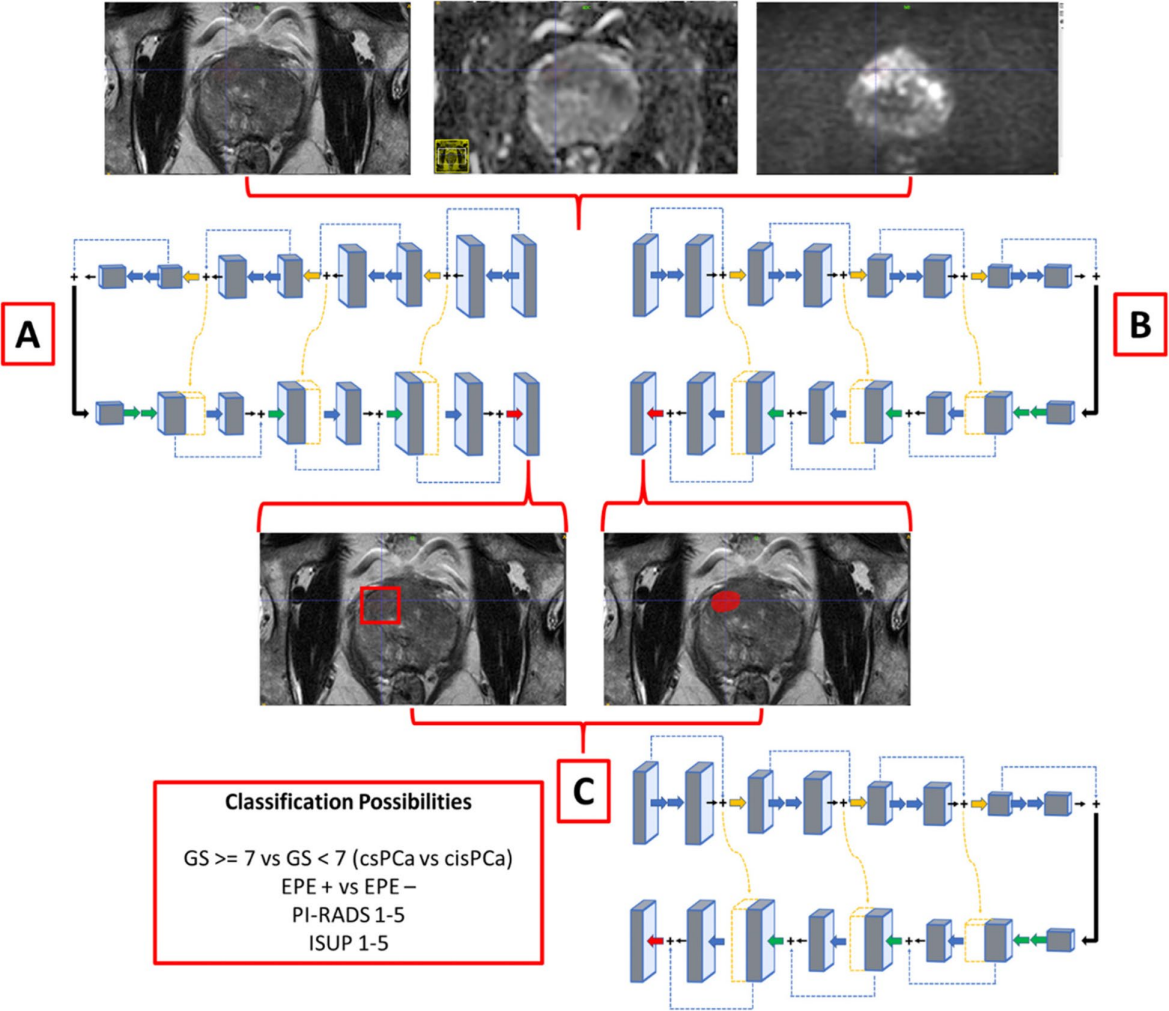
Example of potential artificial intelligence outputs from combining the three sequences of multi-parametric magnetic resonance imaging, with (**A**) representing the detection arm identifying the bounding box, (**B**) representing the lesion segmentation arm, and (**C**) representing the classification arm with the ability to classify lesions from pre-annotated bounding boxes, lesion segmentations, or full non-annotated images. *EPE* Extra-prostatic extension, *GS* Gleason score, *ISUP* International Society of Urological Pathology, *PI-RADS* Prostate Imaging Reporting and Data System

**Fig. 2:**
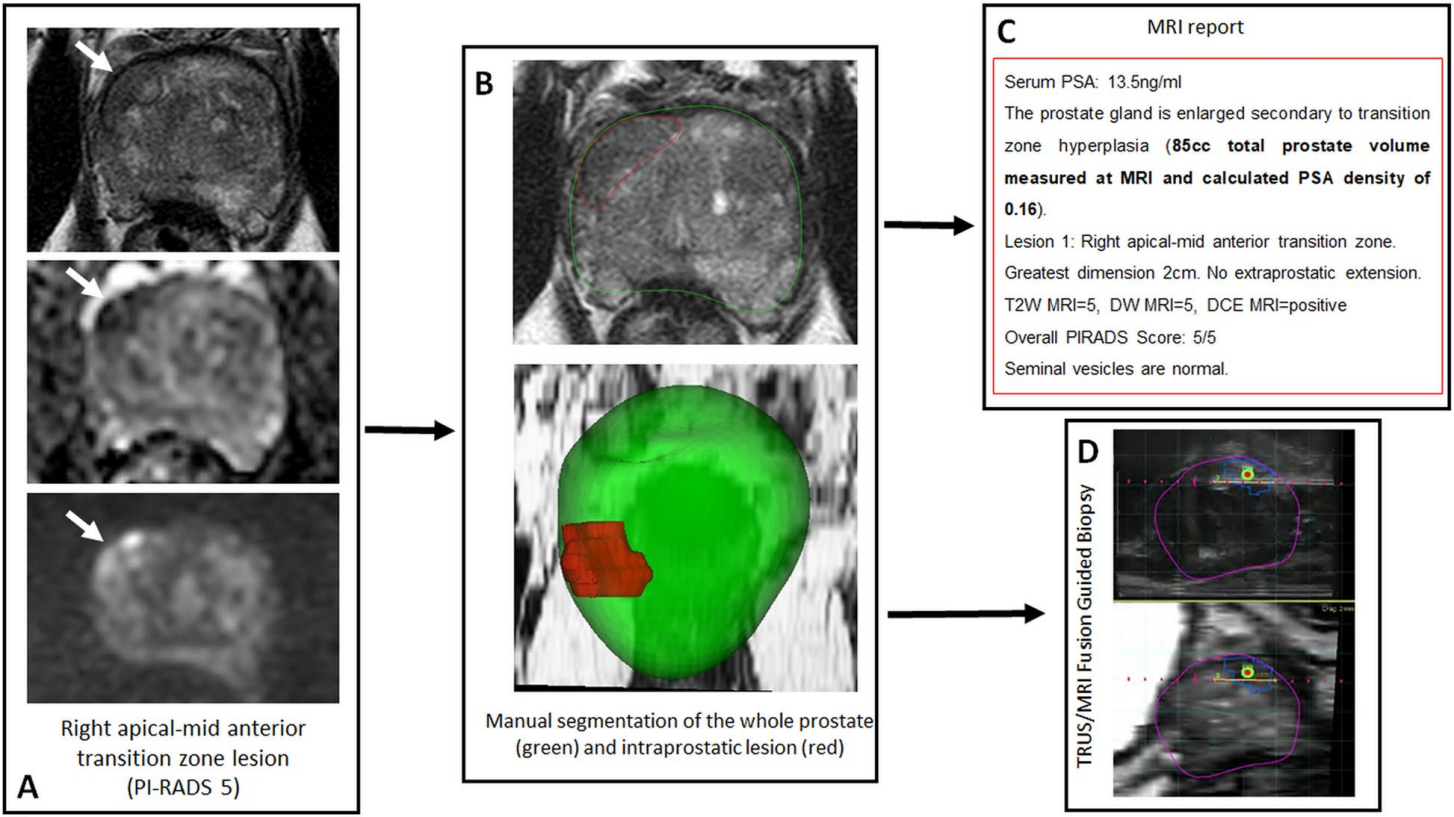
The potential impact of AI-driven prostate segmentation on the workflow of the radiologist. A 6-year-old male patient with a serum PSA of 13.5 ng/mL: multiparametric MRI (T2-weighted sequence, ADC map; and diffusion-weighted image obtained with *b* = 1,500 s/mm^*2*^) (**A**); manual segmentation or AI for total prostate organ and lesion detection/segmentation may be run (**B**); segmentation outputs aid in calculation of variables such as PSA density and greatest lesion dimension and classification (AI can potentially assign PI-RADS scores or predict suspicion of extra-prostatic extension to generate the report (**C**); output segmentations can be used for registration to ultrasound for TRUS/MRI fusion guided biopsy (**D**). *AI* Artificial intelligence, *ADC* Apparent diffusion coefficient, *MRI* Magnetic resonace imaging, *PI-RADS* Prostate Imaging Reporting and Data System, *PSA* Prostate-specific antigen, *TRUS* Transrectal ultrasound

In this narrative review, we introduce emerging medical imaging AI paper quality metrics such as the Checklist for Artificial Intelligence in Medical Imaging (CLAIM) and Field-Weighted Citation Impact (FWCI), dive into some of the top AI models for segmentation, detection, and classification (Fig. 1), and also mention potential areas of impact in the radiologist workflow (Fig. 2).

### CLAIM and FWCI

CLAIM was developed in 2020 [10] to aid authors in presenting research and reviewers in reviewing already published AI manuscripts in medical imaging. The CLAIM checklist, modified after the Standards for Reporting of Diagnostic Accuracy Studies, STARD, guidelines, was specifically designed to address applications of AI in medical imaging that include classification, detection, reconstruction, and workflow optimization. The checklist consists of 42 criteria that should be considered or viewed as “best practice” for presenting medical imaging AI research [10]. This CLAIM checklist can easily be turned into a percentage of CLAIM fulfillment, an objective assessment based on if a paper reports in a way that is considered “best practice” via fulfilling the applicable CLAIM requirements *versus* if they do not.

FWCI, a common Scopus article metric, is the ratio of the total citations received by the total citations that would be expected based on the average of the subject field. A FWCI of 1 means that the paper performs just as expected for the global average whereas more than 1 means that the paper is more cited than expected according to the global average (a FWCI of 1.48 means 48% more cited than expected) and less than 1 means that the paper is cited less than expected according to the global average. Both CLAIM and FWCI can both be used as markers as of article/research impact and rigor and are encouraged to be used in AI manuscript reporting and evaluation.

Overall, there were 29 classification/detection papers: 18 detection papers, 4 detection and classification papers, and 2 which did not indicate. When comparing papers that created classification models (*n* = 29) *versus* those that created detection models (*n* = 18), the mean AUC was 0.843 for classification models (*n* = 25 of 29 reporting) and 0.832 for detection models (*n* = 15 of 18 reporting), while the mean field-weighted impact factor was 4.79 for classification models (*n* = 26 of 29 reporting) and 3.64 for detection models (*n* = 18 of 18 reporting), and the mean CLAIM percentage fulfillment was 77.8% (*n* = 29 of 29 reporting) for classification papers and 71.2% for detection papers (*n* = 18 of 18 reporting).

The classification and detection papers that were in the top 25% with respect to field-weighted impact factor of the sampled papers and those which have the largest sample sizes and highest CLAIM percentage fulfillment are discussed below as we believe these papers will represent the most impactful and potentially generalizable. Additionally, emerging segmentation AI papers are also introduced which were not covered in this prior review (Table 1).

**Table 1:** Summary of the artificial intelligence (AI) development papers discussed in detail

First author [reference number]	Overall sample size	AI family	AI method	Public/external datasets used	Images used	Loss functions	AUC	Dice similarity coefficient
Wang [11]	90	Whole gland segmentation	3D CNN + skip connections	PROMISE12	T2-weighted	Cross-entropy + cosine loss		0.86−0.88
Ushinsky [12]	299	Whole gland segmentation	Hybrid 2D-3D CNN + skip connections		T2-weighted	Adam loss		0.88
Sanford [13]	648	Whole gland segmentation	Hybrid 2D-3D CNN	Five separate unaffiliated institutional independent datasets	T2-weighted	Dice similarity coefficient loss		0.931
Cao et al. [14]	417	Lesion detection	3D CNN FocalNet		T2-weighted, ADC maps, echo-planar	Mutual finding loss	0.81	
Ishioka [15]	335	Lesion detection	U-net + ResNet50 (skip connections)		T2-weighted	Adam loss	0.64–0.65	
Le [16]	364	Lesion classification	Two parallel 2D CNNs	The Cancer Imaging Database (TCIA)	T2, ADC maps	Similarity loss	0.91	
Liu et al. [17]	341	Lesion classification	3D CNN XmasNet	PROSTATE-x	T2, ADC, diffusion-weighted, *K*_trans_	Adam loss	0.84	

*2D* Two-dimensional, *3D* Three-dimensional, *ADC* Apparent diffusion coefficient, *AUC* Area under the curve, *CNN* Convolutional neural network

### AI-based prostate segmentation

**Fig. 3:**
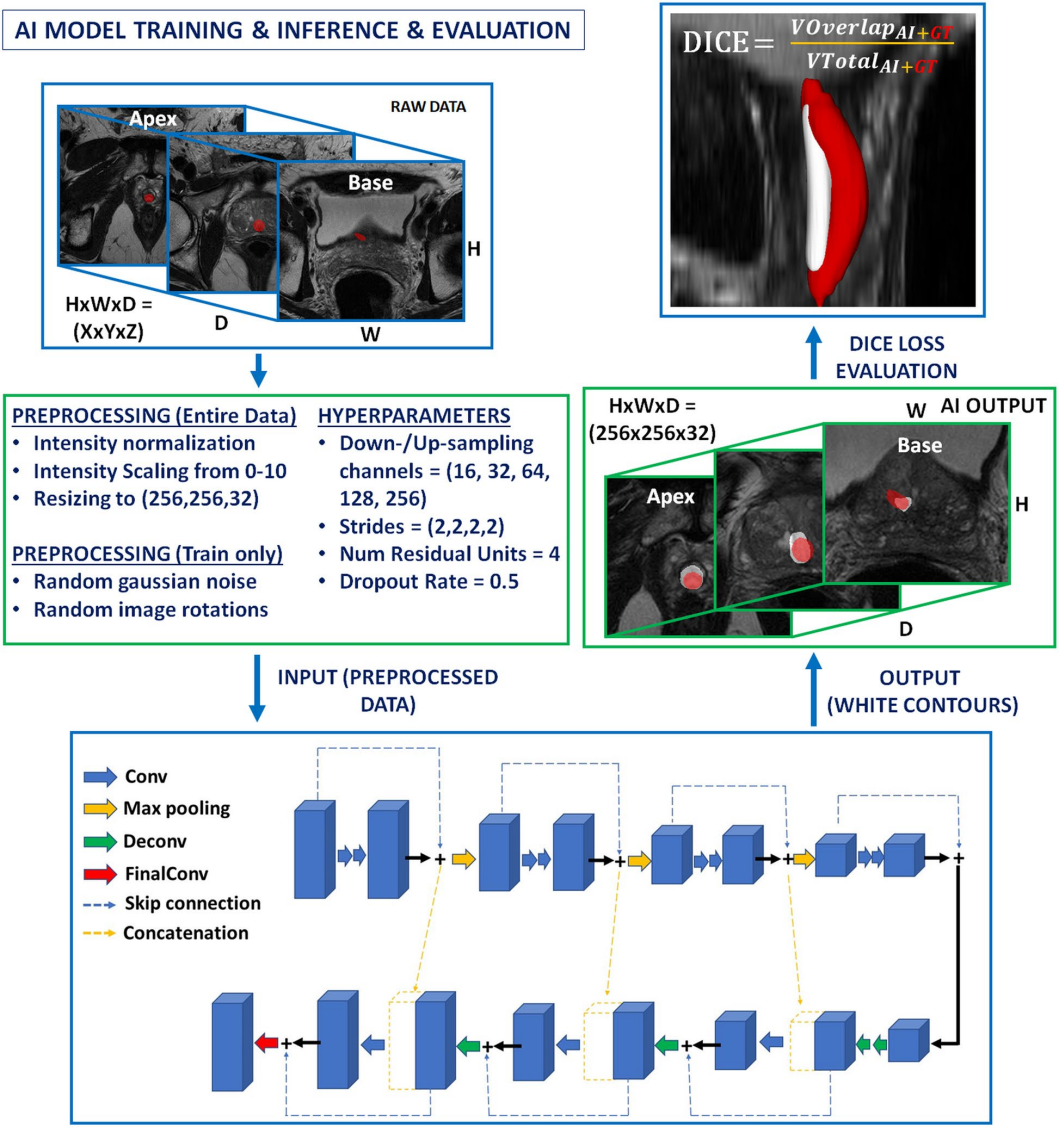
Typical training/inference/evaluation workflow of segmentation AI showing a three-dimensional CNN U-Net for automated segmentation of the prostatic urethra. First, the raw T2-weighted image undergoes preprocessing for intensity normalization, size scaling/cropping, and for training the image undergoes additional data augmentations. Second, the preprocessed image is fed into the CNN which outputs the prediction or white segmentation. Third, the ground truth red urethral contour is compared to the AI-predicted white contour, the loss or difference is computed, and this loss is communicated back to the CNN for tuning of neuronal weights. At the final stage, the performance evaluation of the AI model is conducted using the Dice similarity coefficient. *AI* Artificial intelligence, *CNN* Convolutional neural network

Prostate segmentation AI is developed to extract out anatomical/lesion regions-of-interest similarly to manual segmentation but attempts to address the variability of segmentations that result from readers of different experiences and MRI scans of varying quality [18]. A typical anatomical AI segmentation training/inference workflow is shown in Fig. 3 from a prostatic urethra segmentation AI model [19]. Segmentation AI will attempt to output the exact outline of the desired object/volume of interest (Fig. 1b). Segmentation of the prostate and its related structures is very important for identifying its capsule, prostatic zones, urethra tract, and intraprostatic lesion locations. Identification of these areas allows for improved the treatment of benign prostate hyperplasia, surgical and targeted-biopsy planning, radiotherapy dosage/toxicity calculations, and predicting cancer-specific survival and prognosis [5, 20].

Segmentation of prostate MRI has critical clinical uses such as accurate estimation of the entire prostate gland volume for calculating the serum prostate-specific antigen (PSA) density and MRI data preparation for biopsy guidance in transrectal ultrasound/MRI fusion guided biopsy systems and for radiotherapy planning. Manual segmentation of the prostate and its sub-organs such as the urethra is a time-consuming task and is very much prone to interoperator variations [21]. AI has been commonly used for prostate segmentation and currently there are few commercial solutions for this time-consuming process [7].

Recently, DL-based AI solutions are reported commonly to provide robust performance for segmenting the prostate gland and its zones. In a study by Wang et al. [11], a three-dimensional (3D) fully convolutional network with deep supervision was used to develop a fully automated prostate segmentation model for T2- weighted MRI. The authors reported a mean Dice similarity coefficient of 0.88 (range 0.83−0.93) between AI model and manual segmentations for the whole prostate. Wang et al. [11] utilized a *combined loss function* of both *cross-entropy loss* and *cosine loss* in order to take advantage of their individual strengths and attempt to achieve better quantitative and qualitative performance. The cross-entropy loss is generally optimized for voxel-level accuracy, while other loss functions such as cosine similarity loss are helpful for improving the segmentation quality. In another study, Ushinsky et al. [12] developed a hybrid 3D-two dimensional (2D) U-net based segmentation algorithm for automatic localization and segmentation of prostate gland at T2-weighted MRI of 299 patients. The AI-based whole prostate segmentation model achieved a mean Dice similarity coefficient of 0.898 (range, 0.890–0.908) when compared with manual segmentations. The model Ushinksy et al. developed leverages features from multiple axial slices simultaneously to better construct a single 2D image. They say this architecture imitates a radiologist who will typically interpret multiple axial images before making decisions on one 2D image. Finally, in a study by Sanford et al. [13], a DL approach combining 2D and 3D architectures with transfer learning incorporation was used to develop a whole prostate and transition zone segmentation algorithm in 648 patients. The study reported mean Dice similarity coefficients of 0.931 and 0.89 for whole prostate and transition zone, respectively. This study utilized a data augmentation strategy which was specific to the gland deformations, intensity variations and alterations in image acquisition for MRI data from five different centers and this novel strategy improved the whole prostate and transition zone segmentation performances 2.2% and 3%, respectively. Prostate segmentation AI is the most studied part of prostate MRI workflow and current research indicates that 3D DL-based applications can offer state of art solutions for this time-consuming task during prostate MRI read out and biopsy planning for radiologists. Figure 3 illustrates how segmentation AI might improve and supplement the workflow of a radiologist. Many of these strategies discussed above for anatomical segmentation of the prostate and its sub-organs also apply to suspicious lesion segmentation as well.

### Intraprostatic lesion AI detection

AI for prostate cancer detection is mainly used to identify cancer suspicious areas within a prostate MRI scan and do not require prior lesion annotation by radiologists [8]. AI-based detection models may range from two-class lesion detection (csPCa *versus* cisPCa) systems to multiclass lesion detection systems such as the International Society of Urological Pathology (ISUP) score [22] or the Prostate Imaging Reporting and Data System (PI-RADS) score [16]. In contrast to segmentation, which generally provides the exact outline of an object within an image, AI-based detection helps to create bounding boxes around suspicious objects (Fig. 1a). To date, several studies have evaluated AI algorithms developed for prostate cancer detection on mpMRI. Despite the many differences in feature extraction, MRI techniques, and study populations, these studies demonstrate a robust detection rate: 75 to 80% or more. Notably, this is within the range of reported radiologist performance [1].

For AI-based detection there have been several validation studies investigating if these AI truly have an impact on the radiologist workflow. In a recent multireader, multi-institutional study, Gaur et al. [23] showed that AI-based detection improved specificity when combined with PI-RADS v2 [24] categorization. This AI-based detection also slightly improved radiologist efficiency and found an index lesion sensitivity for PIRADS v2 ≥ 3 of 78% [23]. Litjens et al. [25] and Song et al. [26] have also demonstrated the improved detection of cancer and discrimination of csPCa from cisPCa when combining AI-based prediction and PI-RADS v2 [25, 26].

**Fig. 4:**
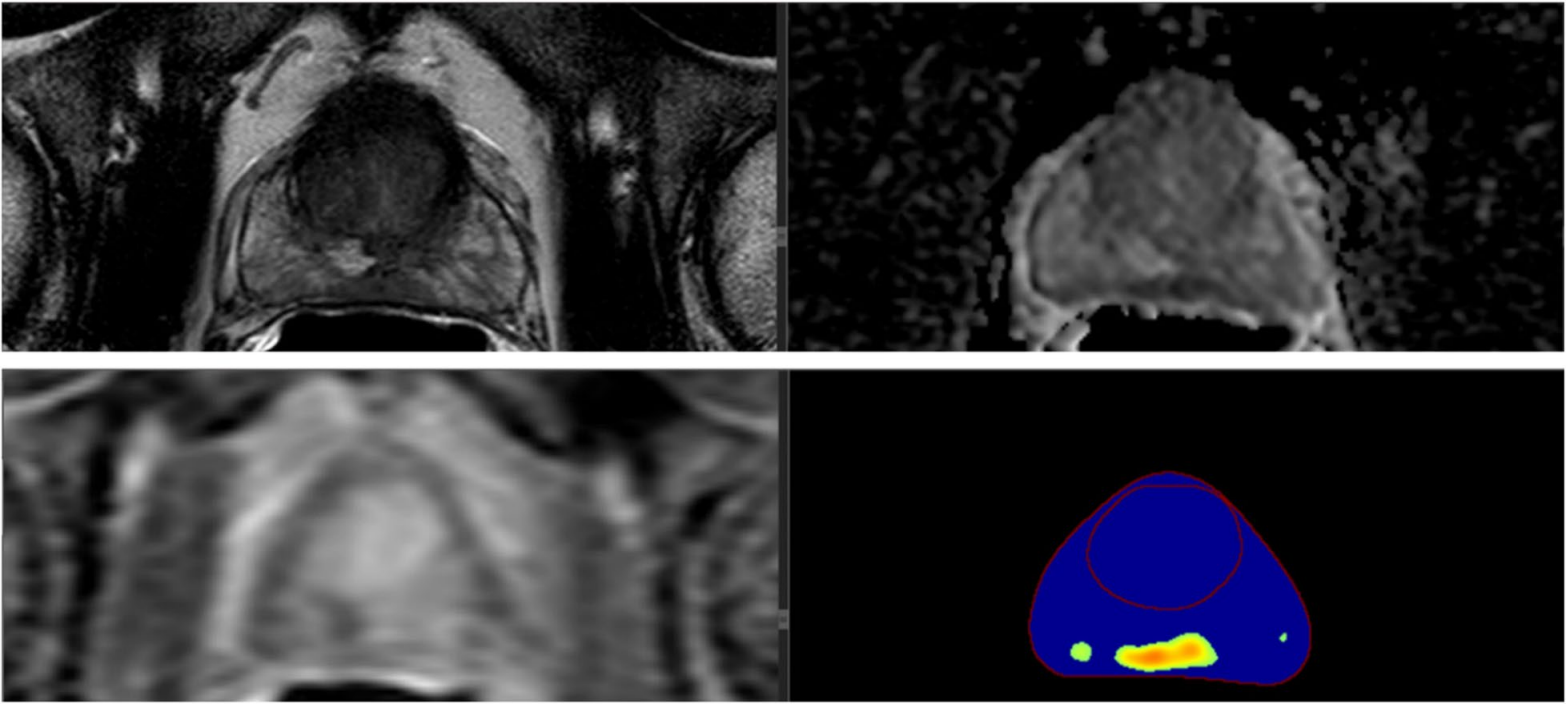
Multi-channel prostate lesion computer-assisted detection model outputting probability maps and contours of prostate zones for interpretability

The top AI studies developing detection AI are further discussed. One DL paper with a CLAIM percentage fulfillment of 68.3% and a FWI of 6.04 by Cao et al. [14] developed a joint prostate cancer detection and Gleason score prediction model on a dataset of 417 patients who underwent mpMRI [14]. The model combined T2-weighted turbo spin-echo imaging and maps of the apparent diffusion coefficient (ADC) using diffusion-weighted echo-planar imaging and stacked them as different imaging channels before feeding into FocalNet, an end-to-end multi-class CNN. One unique addition that Cao et al. [14] made is what they call *mutual finding loss*. It tries to address the challenge that different components of mpMRI (T2-weighted and diffusion weighted sequences, ADC maps, dynamic contrast-enhanced sequences) capture distinct information and only a portion of the information is shared across all components when stacked in a multichannel AI detection (Fig. 1a). As a result of this, findings which are observable in one component may be partially observable or non-observable in the other components. During the end-to-end AI model training, a CNN with stacked components as proposed by Cao et al. [14] can learn the common features across components, effectively emulating the normal process of a radiologist’s reading mpMRI, based on a combination of the various imaging findings on the subcomponents of mpMRI. For the detection of histopathology-proven index lesions and clinically significant lesions, their FocalNet achieved 89.7% and 87.9% sensitivity at one false positive per patient and showed a sensitivity only 3.4% and 1.5% lower than that of experienced radiologists using PI-RADS v2 [14]. Another DL detection paper with a CLAIM percentage fulfillment of 75% and FWI of 7.69 by Ishioka et al. [15] shows the power of AI network ensembling by combining a U-net with ResNet50 and introduces neural network interpretability and probability maps. U-net has the potential to distinguish whole and local pelvic structures and ResNet can then reformulate the CNN layers to learn them as residual functions instead of learning unreferenced functions. It is generally understood that residual functions help to eliminate the vanishing gradient problem in AI by allowing it to communicate with intermediate CNN layers. One unique contribution from Ishioka et al. [15] is the visualization of the feature and probability maps within the CNN as a way of interpreting which imaging features the AI is using the most for predictions [15]. These feature maps are paramount in attempting to explain the logical structure of a neural network, which is often expressed as a “black box” and may take form as feature maps, saliency maps, or probability maps [see Fig. 4 showing probability maps for AI detection]. Overall, intraprostatic lesion detection is one of the most critical steps of prostate MRI read outs and it requires significant expertise and is prone to interobserver variation commonly. Currently, quite a few research-based AI models exist for this task however, to document their actual impact on improving clinical management, which is mainly including biopsy decisions, prospective and multicenter studies are needed.

### Intraprostatic lesion AI classification

Intraprostatic lesion classification AI models are used to classify either full images or to classify preannotated regions-of-interest ranging from two classes (csPCa *versus* cisPCa) to multiple separate classes (histopathological grading also known as ISUP score or PI-RADS score) as seen in Fig. 1. AI lesion classification typically does not perform voxel-level predictions but is commonly entire image/region-of-interest based. Patients with cisPCa are those with ISUP 2 or lower and are usually eligible for *active surveillance* whereas men with higher grade lesions such ISUP greater than 2 are typically advised to undergo active treatment such as focal therapy, radical prostatectomy, or radiotherapy [8].

Accurate lesion classification is important for selecting appropriate management options as any one therapy has a mosaic of side effects. Reductions in unnecessary biopsies is important in preventing common biopsy complications including infections, hematuria, rectal bleeding, hematospermia, lower urinary tract symptoms, and temporary erectile dysfunction [4]. Suarez-Ibarrola et al. [2] found within the literature that lesion classification accuracy of the algorithms they looked at was comparable to that provided by radiologists using PI-RADS [2]. An original research study [16] with a percentage CLAIM fulfillment of 68.3% and a FWI of 6.81 developed a classification CNN for (i) detecting cancerous *versus* noncancerous lesions and (ii) differentiating csPCa *versus* indolent cisPCa. This paper designed a new similarity loss function like mutual finding loss utilized by Cao et al. [23], allowing for the fusion of common and consistent features from ADC maps and T2-weighed images. This allows the CNN to “see” the true visual patterns of PCa across the spectrum of imaging sequences. Otherwise, without similarity loss functions, imaging features from different mpMRI sequences such as those being T2-weighted will not be able to fill in the information gaps that arise from other functional sequences such as ADC maps, diffusion-weighted images, or vice versa.

Le et al. [16] then combined the classification results of the multimodal CNN with results based on hand-crafted features using a support vector machine classifier. Experimental results from an extensive clinical dataset from 364 patients with a total of 463 PCa lesions and 450 noncancerous lesions demonstrate that their system can achieve a sensitivity of 89.9% and a specificity of 95.8% for distinguishing cancerous from noncancerous tissues. With respect to csPCa *versus* cisPCa, they achieved a sensitivity of 100.0% and a specificity of 76.9%. This paper also demonstrated superior performance compared to the state-of-the-art method relying on handcrafted features alone [16]. Another original research study [17] with a percentage CLAIM fulfillment of 70.6% and an FWI of 23.12 developed XmasNet, a novel deep learning architecture based on CNNs, for classification of prostate lesions on MRI. This study showed that with the strength of data augmentation via 3D rotations and slicing, their XmasNet outperformed traditional ML models based on engineered features. Their XmasNet outperformed 69 methods from 33 participating groups and had the second highest AUC (0.84) in the 2017 PROSTATEx challenge. Like intraprostatic lesion detection AI models, several research-based AI algorithms are defined for intraprostatic lesion classification task and further research is needed to depict the benefit of these AI models in radiologists’ performance.

### Moving forward with prospective AI ptudies

AI can potentially play a major role in further improving prostate MRI contribution to the clinical management of localized PCa. The majority of the work in prostate MRI AI reveals promising results for various tasks of prostate MRI interpretation and data processing for biopsy; however quite a limited amount of this work has reached to actual translation phase to clinics so that a clear benefit of AI on prostate MRI workflow is yet to be demonstrated. For this to happen, one of the “musts” is proving the benefit(s) of AI in prospective clinical trials. The potential benefits can be listed as improved performance in comparison with radiologists’ and reduction in read out times and inter-reader variation. Moving forward comfortably with AI will require this critical prospective multicenter evaluation.

## Conclusions

AI is a commonly studied topic for prostate MRI and several groups report AI models for prostate segmentation, intraprostatic lesion detection and classification tasks with promising results. Some of the best models across the applications discussed utilize 3D AI models and special loss functions that attempt to combine the findings and fill in gaps introduced by different mpMRI sequences. Prospective studies with multi-center design will be needed to depict the impact of AI on radiologist performance and clinical management of prostate cancer.

## Abbreviations

2D: Two-dimensional
3D: Three-dimensional
ADC: Apparent diffusion coefficient
AI: Artificial intelligence
AUC: Area under the curve
cisPCa: Clinically insignificant prostate cancer
CLAIM: Checklist for Artificial Intelligence in Medical Imaging
CNN: Convolutional neural network
csPCa: Clinically significant prostate cancer
DL: Deep learning
FWCI: Field-weighted citation impact
ISUP: International Society of Urological Pathology
ML: Machine learning
mpMRI: Multiparametric magnetic resonance imaging
MRI: Magnetic resonance imaging
PCa: Prostate cancer
PI-RADS: Prostate Imaging Reporting and Data System
PSA: Prostate-specific antigen
